# A picture is worth a thousand words: A proposal to incorporate video into the evaluation of adults with intellectual or developmental disability living outside the home

**DOI:** 10.3389/fpubh.2022.887714

**Published:** 2022-08-24

**Authors:** Briana C. Prager, Sherri M. Broder, Marvin R. Natowicz

**Affiliations:** ^1^Cleveland Clinic Lerner College of Medicine, Cleveland Clinic, Cleveland, OH, United States; ^2^Medical Scientist Training Program, Case Western School of Medicine, Cleveland, OH, United States; ^3^Department of Bioethics, Case Western Reserve University, Cleveland, OH, United States; ^4^Pathology and Laboratory Medicine, Genomic Medicine, Neurological and Pediatrics Institutes, Cleveland Clinic, Cleveland, OH, United States

**Keywords:** disability evaluation, developmental disability, adults with intellectual or developmental disabilities, intellectual disability, health disparity, health policy, vulnerable population, video evaluation

## Abstract

Adults with intellectual or developmental disability (IDD) comprise 1–2% of the population worldwide. IDD is a significant risk factor for premature morbidity or mortality. This is likely due in part to preventable health conditions, which are modifiable with the intervention of direct care providers in areas including nutrition, promotion of an active lifestyle and effective identification of health or functional deterioration. Adults with IDD are also at increased risk for neglect or mistreatment, a finding that has been documented across multiple countries and in a variety of care settings. Contributing factors include resource availability, lack of person-centered care, management culture and care worker training. Practical and economical interventions may address the known disparities and challenges facing the large community of adults with IDD. To promote person-centered care, improve record-keeping/documentation, and aid in protecting the health and safety of this vulnerable population, we propose incorporation of a video into the evaluation of adults with IDD living outside the home.

## Introduction

Adults with intellectual or developmental disability (IDD) comprise 1–2% of the population worldwide and over 4 million people living in the United States (1, 2). These individuals are at increased risk for premature morbidity or mortality associated with preventable illness or deterioration (3–11) and have a 20-year decrease in life expectancy (12). Adults with IDD are also at risk for neglect and mistreatment, a finding documented across multiple countries and in a variety of care settings (13–16); key contributing factors have been identified (13, 17–21). Thus, adults with IDD represent a large and vulnerable population that experiences disparities in health and overall well-being.

Stakeholders in the system including service users and their families, care workers and case workers, face significant challenges in safeguarding the well-being of adults with IDD. Protective factors include positive attitudes of the staff toward the residents, value congruence and the perception of a relationship [reviewed in Collins and Murphy, 2021] (13, 19, 22). Conversely, a perception of ‘otherness', ‘resistance' or an attitude of ‘staff-centeredness' were associated with worse care performance (18). Care workers in under-performing living situations were more likely to consider the service user as ‘too disabled' or lacking skills (23); in some instances, individuals with IDD may not even be considered as persons. Frequent staff changes may also impede the development of long-term, trusting relationships between staff and service users (24). In a large-scale survey of service users, families and care workers, negative factors included a lack of respect for clients, the absence of person-centered practice and insufficient resources for effective oversight (17).

Several strategies have been proposed to enhance protective factors and thus improve the well-being and care of adults with IDD living outside the home. Many of these proposals center around enhancing person-centered practices by care workers through promoting increased knowledge about and positive relationships with the individuals for whom they care. Examples include involving family members in training to increase reciprocal ‘understanding and empathy' (25) and increasing staff awareness of a service user's interests. Person-centered care is at the crux of Medicaid requirements for home and community-based services and emphasizes respecting the individual being cared for, treating that person as a unique individual, understanding their perspective and fostering a positive environment (26, 27). Video-based strategies can effectively increase positive attitudes of viewers toward adults with disabilities or dementia and decrease perception of otherness (28–30). Thus, a video may serve to not only provide information in a way that is convenient and easy to consume, but also generate a narrative framework to stimulate positive perceptions and interactions.

Health care disparities, neglect and mistreatment are facilitated by inconsistent record-keeping and ineffective oversight secondary to a systemic lack of resources, variability in management, and frequent turnover of care workers and of case workers (13, 17, 31). Proposed interventions to improve oversight include the use of constant video surveillance, which has limitations in difficulty of implementation, privacy considerations and service user wishes (32). Augmenting existing evaluation systems with short videos would provide a record of functional and behavioral status and aspects of physical health. This form of documentation would be a non-intrusive, objective method of assessing well-being and health status. Such a record, when viewed at a later time, could also alert case workers or health care providers to declines in well-being or function that may not be otherwise evident, particularly given the frequent transitions of care workers and their associated lack of knowledge of the baseline status of the persons for whom they care (24, 33).

Video-based record keeping and documentation of a functional baseline may also assist the individual's medical team including the primary care physician and other clinicians. Ineffective transmission of health information is a well-documented challenge related to health disparities for adults with IDD (3, 5). The use of short videos has proven to be an inexpensive, commonly used and helpful aide in other contexts such as the evaluation of persons having possible seizures or other paroxysmal events by neurologists (34, 35). Concise video-based records may help establish a historical baseline and facilitate a longitudinal assessment of an individual's functional and health status (3, 5).

For adults with IDD living in residential facilities paid for by government funds, oversight is regulated at the state and county level as well as by federal regulations related to Medicaid funding. States utilize individual service plans (ISPs) to perform intake and repeat assessments of an individual's support needs, functional status and limitations, likes and dislikes, communication style, etc. Regulations surrounding ISPs vary across states and, frequently, counties and differ depending upon an individual's functional status and the required level of service (36). Tools to augment the evaluation process should be convenient, easy-to-use and inexpensive in order to promote effective use and avoid placing excessive burden on caregivers or case workers. We propose incorporating one short video into the evaluation process to provide accessible information about the physical and functional status of service users. These videos would serve to educate care workers and case workers about the adult with IDD they work with, promote a humanistic, positive perception of the service user and provide a baseline record of functional status and well-being to improve oversight and avert neglect.

## Aim and rationale

Premature morbidity and mortality, neglect and mistreatment are much more common in adults with IDD, as detailed above. Systems in place to prevent these occurrences, including ISPs, are incompletely successful at best. ISP documents vary in the extent and quality of coverage of issues that are central to the adults' lives and require reading of the entire, sometimes 80 page, document. Direct care providers come from a diversity of educational, cultural and linguistic backgrounds and not all persons are effective learners through reading; some individuals are better able to learn similar material through visual methods. In addition, the prose descriptions of service users do not usually engender respectful, empathic connections with them. Finally, the prose of a typical ISP does not enable the reader to have a quality image of what the service user looks like and his/her overall state of well-being. That, coupled with the high turnover of direct care workers and case workers of 30–70% per year, means that there is no good official preservation of the nature of a service user's state of appearance and well-being at the time of intake (33, 37). Even in as short a time as 2–3 years, it is unlikely that any of the direct care staff will have firsthand knowledge of the appearance, health and functionality of the adult they are caring for, so-called institutional memory. A short, adjunctive video is an easy-to-create, inexpensive means to overcome these limitations of the ISP.

We propose creating one video during intake when the adult with IDD is considered for placement in a residential facility licensed by state or local authorities and/or paid for by government funds. A short video integrated into the intake process is not intended to replace the ISP; the ISP is a process and document that serves multiple crucial needs relating to the life of the service user. Rather, the proposed video would serve as an essential adjunct to the ISP, addressing some of the service user's needs that are not met by an ISP and duplicating but enhancing some others. High-level aims of the video are to: (1) create a video-based document that enhances the likelihood of adoption of a person-centered approach to the service users' care by direct care and case workers and (2) provide a baseline of selected key aspects of service users' health, well-being and functional status. In order to maximize uptake and utility, the video components should be concise, relatively standardized with flexible but clear guidelines for filming and include a multifaceted assessment that would be useful to a variety of care providers.

## Video content

The content of the video is derived from the aims noted above. Its scope and depth of coverage are limited, however, by the desire to make the film 30 min or less in duration to ensure that it will be viewed. It is also important to recognize the diversity of needs, interests, abilities and disabilities of adults with IDD. This will necessarily be reflected in variability of the topics covered.

To enhance the likelihood of use of a person-centered approach, central elements of the film should include the following: discussion of the individual's interests and likes and dislikes in diverse areas (e.g., foods, recreational and other activities, interpersonal relationships), matters that may upset the service user or cause discomfort and helpful de-escalation techniques if the user becomes agitated.

To provide a baseline of service users' health, well-being and functional status, there should be visual documentation of the adult's general appearance, language and communication skills, visual and auditory function and selected gross and fine motor functions. The video should include details that may be otherwise difficult to convey in a non-visual format that may help evaluate ongoing well-being when viewed longitudinally (e.g., if there has been subsequent loss of subcutaneous fat or of muscle mass, change in facial expression or eye contact, changes in balance or ambulation, changes in fine motor function, etc). Supplementary Table 1 provides examples of these content elements and commentary related to each.

## Production

The video would be filmed by an individual involved in the care or oversight of the adult with IDD, for example a care worker or social worker, and could be recorded with the help of others including a health care worker and a family member or guardian, if possible. Informed consent should be acquired from all parties including the caregiver, parent or guardian and, when possible, the service user. If informed consent can be obtained from the service user, he/she should be allowed to refuse the filming and the care team should respect that position and offer to film at another time. Even if informed consent does not legally need to be provided by the adult with IDD, the process and goals should be explained and feedback solicited to the extent that it is feasible. A clear set of guidelines would be provided to the videographer regarding what to film and how to film. Filming should occur at a site that is familiar and comfortable to the service user, with a family member or guardian present, if possible, and might also include a friend or pet. A person who knows the adult (e.g., a family member, guardian or friend) would provide commentary by answering provided questions regarding the individual's interests, likes/dislikes, needs and health and functional status. The adult with IDD should be included in the discussion of these issues if he/she is able to communicate using verbal language, signs or facial expression. Insofar as the video will contain medical and other personal information, appropriate legally binding agreements between the service user or his/her guardian and (a) persons making the film, (b) any person who is about to view the video and (c) the legal representative of any organization that intends to house the video should be signed to reduce the likelihood of inappropriate distribution or alteration of the content of the video. In addition, technical measures can be implemented to restrict access to and prevent modification of the video such as the use of an application that provides password protection for the video.

## Implementation and evaluation

This proposal could initially be implemented as a pilot project within a subset of facilities or a county in order to evaluate benefits and challenges and optimize the integration process. Ultimately, it would be most effective as a legal requirement overseen by the county or state. The video series could be utilized during care worker training to educate about the individual adult with whom they will be working, by case workers to assess changes in functional status and well-being since the time of the intake and potentially by health care workers. Given the accessibility and ease of use of video editing technologies, we do not anticipate that special skills would be required to film and finalize the video; videos can even be made using cell phones.

Evaluation of the intervention is an important component of any pilot project that aims to be generalizable. While limited tools currently exist to evaluate quality of care in the out-of-home setting for adults with IDD, one promising tool is the Group Home Culture Scale. This scale assesses seven features of the care environment, at least three of which would potentially be influenced by this intervention: (1) the extent to which staff practices are directed toward the well-being of each resident, (2) the extent to which staff value the residents and relationships with them and (3) social distance or ‘otherness' (38). Other important evaluative measures that could also be used include: the number of times the video is viewed and by which parties, both during initial intake and during follow-up sessions, and end user satisfaction with the process, including caregivers, social workers, group home managers, and persons with disability.

## Benefits and challenges

Our proposed video is inexpensive and easy to carry out. A video-based assessment does add another responsibility to the videographer, e.g., a case worker. However, given that the proposed intervention is simple, flexible and inexpensive and may be incorporated into an existing evaluation process, it is unlikely to be significantly burdensome. Intake video assessments would provide objective, visual data to ensure well-being and encourage a person-centered approach. This would be a convenient, easy way for a direct care worker to learn about the adult with whom they are working. Furthermore, visual aids, particularly narrative-based ones, have been shown to facilitate development of empathy and a positive attitude toward others (28, 30), which are protective factors against neglect and abuse (13, 19, 22). Knowledge of an individual's functional capacity and enjoyed activities may allow a direct care provider to provide activities that promote overall mental and physical health. Awareness of their likes and dislikes with respect to food may facilitate meal preparation and thus nutrition. An understanding of the service user's communication style including non-verbal cues, verbal capacity, receptive language function and behavioral triggers may improve relationships between the care provider and service user. This may in turn enhance the social environment for both parties and reduce care provider burnout.

Finally, a video may be highly beneficial to health care workers in establishing a baseline of the health and functional status of an adult with IDD. If the video is effective, future efforts could integrate annual videos into the assessment process. Such a video series would generate a longitudinal visual record of service users' health and well-being.

## Discussion

Adults with IDD living outside the home have multiple vulnerabilities. They face many challenges in securing their well-being, as do the care workers, case workers, families and medical teams who aim to optimize their care. Institutional memory is often lacking due to frequent staff turnover for these vulnerable individuals with disabilities, some of whom lack close family members or others who can look out for them. To address these issues, we propose an inexpensive and readily implementable intervention that incorporates video evaluations into the assessment process of adults with IDD living in out-of-home care environments. These videos would serve to (1) educate staff toward needs and preferences of residents to bolster well-being, (2) document key aspects of the health and capabilities of the adult with IDD at the time of intake to ensure institutional memory, and (3) ‘humanize' the adult with IDD to promote person-centered care.

## Data availability statement

The original contributions presented in the study are included in the article/Supplementary material, further inquiries can be directed to the corresponding author/s.

## Author contributions

BP, SB, and MN contributed to the design, writing, review, and revision of the manuscript. All authors read and approved the submitted version.

## Conflict of interest

The authors declare that the research was conducted in the absence of any commercial or financial relationships that could be construed as a potential conflict of interest.

## Publisher's note

All claims expressed in this article are solely those of the authors and do not necessarily represent those of their affiliated organizations, or those of the publisher, the editors and the reviewers. Any product that may be evaluated in this article, or claim that may be made by its manufacturer, is not guaranteed or endorsed by the publisher.
