# Supplementary material for: A picture is worth a thousand words: A proposal to incorporate video into the evaluation of adults with intellectual or developmental disability living outside the home
Source: Front Public Health. 2022 Aug 24;10:887714. doi: 10.3389/fpubh.2022.887714 (PMC9451026; doi:10.3389/fpubh.2022.887714)
Supplement: Supplementary file 1 [file Table_1.DOCX]

Supplementary Material

| **CONTENT** | **KEY ISSUES** | **SPECIFIC COMMENTS** | **GUIDING QUESTIONS** |
| --- | --- | --- | --- |
| Identification of all participants, including age/date of birth of the adult, and date/place of filming. Brief statement of purpose of the film. | Begin to establish personhood using kind introduction to the adult and demonstrating meaningful links with others in his/her life. | Allow the individual who is the subject of the film to introduce him/herself, if possible | Hi, (NAME), we’re here to make a film about you. Can you tell us about yourself? |
| Appearance #1 | Facial features, including facial expression | Include multiple times during the filming so as to capture the range of facial expression. |  |
| Communication | Provide opportunity to learn the various means of communication used by the adult (eg, verbally, facial expression, pointing, signing, gesturing, augmentative communication device) | Learning how the adult indicates interests/needs & discomfort is essential. Commentary by someone who knows the adult well will be helpful. | Hi, (NAME), can you tell me about your favorite things to do? Or, what are your favorite songs? Can we sing together?  Or, can we read together? Or, what are some of your favorite things to do?  Note: If the adult isn’t able to readily communicate these matters, see below at bottom. |
| Personality/behavior |  | See above. It is important to emphasize his/her strengths and connections to others (eg, family/friends). Commentary by those who know the individual well is important. It is also important to learn about helpful de-escalation techniques. | What makes you happy, (NAME)? What are your favorite foods/items/games/other activities? Who are your favorite people?  Note: If the adult isn’t able to readily communicate these matters, see below at bottom. |
| Gross and fine motor function | Ambulation, with or without assistance, if applicable. For non-ambulatory individuals, one should try to film current use of arms and legs.  Hand grip and handedness, if applicable. | Commentary by someone who knows the adult well will be helpful. | Hi, (NAME). Could you walk a little bit (or play catch or other gross motor activity) with me? Note: Most games also involve use of fine motor functions that can be documented at the same time. |
| Sensory | Vision  Hearing  Tactile  Pain | While talking with the adult, one can present a familiar or interesting object in different visual fields, providing information about visual acuity, visual tracking.    One can usually assess the quality of hearing to routine voice when talking to the adult and then seeing if he/she responds to sound.  Commentary by someone who knows the adult well will be helpful. | Hi, (NAME), can you play _____ with me? (Choose activities that will involve use of near and distance vision. This can be done as part of the gross and fine motor activities noted above).  One should also inquire of the adult or of his parents or guardian: if there are types of sounds that are poorly tolerated (eg, ambulance sirens, vacuums, off-key singing); if there are tactile stimuli that are poorly tolerated (eg, tags on necklines of shirts, certain textures); and if there is unusual hyper- or hyposensitivity to pain. |
| Oromotor function | Provide opportunity for the individual to eat and drink favorite items, if the individual obtains nutrition orally. | If done, this provides information on eye-hand and aspects of fine motor coordination as the individual reaches for the food/drink, as well as regarding chewing/swallowing. One should document if there is a history or risk of choking and, if so, specify choke precautions. Inquiry about food allergies is important, even though this should be in the medical record. | Hi, (NAME), would you like something to drink/eat?  Are there any foods or drinks that you can’t eat because of swallowing difficulties or due to allergies? |
| Appearance #2 | General physique | With the individual partially undressed, if feasible, his/her skin, muscle mass and subcutaneous fat can be appreciated and, possibly, abnormalities of the limbs or torso. | Parents or guardian might ask the adult if it’s okay to change his/her shirt. Alternatively, the adult might wear shorts and/or a short sleeve shirt that would enable non-intrusive viewing of the extremities. |
| Conclusion |  | Provide thanks to the individual who is the subject of the film. |  |
| Possible Additional Conclusion – to be included if the adult with IDD is not comfortable or able to discuss the issues at right. These matters can then be addressed by those who know him/her best. | Possible discussion of key information that the adult with IDD may not be comfortable hearing. | Discussion by family member(s), guardian and/or caregiver without the adult who has IDD present. | How does he/she indicate what he/she needs? How do you know when he/she is happy? Can he/she tell you if he/she is hot or cold or tired or hungry? What words or behaviors will let caregivers know that the individual is upset, anxious or sick? How much receptive language does the individual have? What people are important to him/her? What are his/her favorite activities? What are favorite foods/items/songs? Is routine important to him/her? What helps to calm him/her when he/she is upset? What helps to keep him/her from becoming self-injurious or aggressive to others? What are his/her most important strengths? |

**Supplementary table: Video content guide.**

**(1)** This film is to provide general information about the adult’s appearance/well-being, communication skills, certain gross and fine motor functions, and information about some of his/her likes/dislikes, favorite activities/people/pets and compellingly promote person-centered care. It should be understood that this film is especially for use by care workers but many others will benefit from it as well. All users will be able to appreciate the personhood of those who are filmed, their communication skills, appearance and some important gross and fine motor skills as well as possible changes in these areas or personality or behavior over time. Some specialist viewers, such as neurologists, can also glean important information pertinent to their specialty that will be largely ‘invisible’ to other viewers.

**(2)** Adults with IDD are an intellectually, behaviorally and physically extremely heterogeneous group. In view of this, there is no uniform set of activities that can be asked of all of the persons who will be filmed. Thus, asking someone who is able to walk independently is a powerful way to assess multiple neurological functions but this task cannot be asked of someone who is non-ambulatory. Consequently, the videographer should consult in advance with a person(s) who is familiar with the adult to be filmed so that the most appropriate and informative activities will be filmed. The above list of content topics also is not a specification of the order of content to be filmed. Importantly, multiple content items can be effectively covered simultaneously, enabling efficient coverage of the content matter in a short time frame. For example, viewers can simultaneously appreciate facial features/expression and communication, visual and hearing function while watching the adult in conversation. Or, when filming individuals who are walking one can simultaneously learn about their balance, gross motor coordination, possible involuntary movements and more.

**(3)** The adults with IDD should be treated and spoken with respectfully throughout the filming. They should be prepared in advance regarding the purpose of the film and that this will include conversation and involvement in some activities. The filming should last no longer than 30 minutes and done in a setting that is familiar and comfortable for person to be filmed. He/she should be accompanied by a family member, guardian or friend, if possible, as well as by a pet or objects that he/she likes or are calming, if desired.

**(4)** Many individuals with intellectual disabilities have substantial receptive language function, even if they are non-verbal, and a subset of these individuals do not like to be present when they are talked about. It is important to be mindful of this. Family members, guardian and caregivers of such individuals may convey critical information in the absence of the individual, as long as he/she is present for most of the video.
